# The Efficacy of Nicotinamide in Maintaining Mean Deviation in Glaucoma: A Systematic Review

**DOI:** 10.7759/cureus.110196

**Published:** 2026-06-03

**Authors:** Sam Sherratt-Mayhew, Max Bisp, Charles Page, Wing Sze Vangie Koo

**Affiliations:** 1 Ophthalmology, Sandwell and West Birmingham Hospitals NHS Trust, Birmingham, GBR; 2 Internal Medicine, Russells Hall Hospital, Dudley, GBR; 3 Anesthesiology, Sandwell and West Birmingham Hospitals NHS Trust, Birmingham, GBR; 4 Nephrology, Heartlands Hospital, Birmingham, GBR

**Keywords:** glaucoma, mean deviation, neuroprotection, nicotinamide, systematic review

## Abstract

Glaucoma is a major cause of blindness worldwide and is characterized by progressive retinal ganglion cell loss and visual field deterioration. Although intraocular pressure reduction remains the cornerstone of management, disease progression may occur despite optimal treatment. Nicotinamide, a precursor to nicotinamide adenine dinucleotide, has emerged as a potential neuroprotective therapy due to its role in mitochondrial function and cellular energy metabolism. This systematic review evaluated the efficacy of nicotinamide in preserving visual field mean deviation (MD) in adults with glaucoma, alongside its safety profile and broader ophthalmological outcomes. This systematic review was conducted in accordance with the PRISMA guidelines. MEDLINE, Embase, ClinicalTrials.gov, and the Cochrane Library were searched in March 2026. Randomized controlled trials (RCTs) involving adults with glaucoma receiving nicotinamide-containing interventions and reporting MD outcomes were included. Data extraction and risk of bias assessment using the Risk of Bias 2 tool were performed independently by two reviewers. Certainty of evidence was assessed using the Grading of Recommendations Assessment, Development and Evaluation (GRADE) approach. Due to heterogeneity in study design, interventions, follow-up duration, and outcome reporting, meta-analysis was not feasible, and findings were synthesized narratively. Three phase II RCTs involving 152 participants met the inclusion criteria. None of the studies demonstrated a statistically significant improvement in MD following nicotinamide therapy. One crossover study showed nonsignificant improvements in MD with nicotinamide, with a mean improvement of +0.10 dB compared to placebo over 24 weeks. However, all studies met their primary outcomes. Two crossover trials reported significant improvements in electroretinography parameters, and a parallel-arm study reported a significantly greater number of improved visual field test locations. No serious adverse events were reported. Overall certainty of evidence was low due to small sample sizes, short follow-up durations, and methodological limitations. Current evidence does not support nicotinamide for improving MD in adults with glaucoma in clinical practice. Although preliminary findings suggest potential neuroprotective effects, the evidence remains limited and hypothesis-generating. Larger, adequately powered phase III trials with longer follow-up are required before nicotinamide’s neuroprotective efficacy can be determined with validity.

## Introduction and background

Glaucoma is a leading cause of irreversible blindness worldwide. It comprises a group of progressive optic neuropathies, most commonly primary open-angle glaucoma, characterized by degeneration of retinal ganglion cells (RGCs) [1]. Clinically, this results in patients developing gradual peripheral visual field loss with eventual central vision impairment. Although elevated intraocular pressure (IOP), typically due to impaired aqueous humor outflow, is the major known risk factor for progression, the disease often progresses despite optimal IOP control. Standard management focuses on lowering IOP through pharmacological, laser, and surgical interventions; however, these approaches do not fully halt disease progression [2].

The persistence of visual field deterioration despite optimal IOP control highlights the multifactorial, neurodegenerative nature of glaucoma. Mechanisms, including mitochondrial dysfunction, oxidative stress, impaired axonal transport, and excitotoxicity, contribute to RGC apoptosis. As RGCs are postmitotic, their loss is irreversible, underscoring the need for therapies targeting neuronal survival. Neuroprotection has therefore emerged as a promising strategy, aiming to preserve RGC function by modulating shared pathways of cellular injury and apoptosis rather than focusing solely on IOP reduction [2].

Nicotinamide has gained attention as a potential neuroprotective agent, particularly at high doses above supplementary levels, due to its role in cellular energy metabolism and mitochondrial function. It is a precursor to nicotinamide adenine dinucleotide (NAD+), a key coenzyme in oxidative phosphorylation and adenosine triphosphate production. Reduced serum NAD+ levels have been shown in people with glaucoma [3,4]. Preclinical studies show that nicotinamide supplementation can restore NAD+ levels, enhance mitochondrial resilience, reduce oxidative stress, and improve RGC survival [5]. Early clinical evidence suggests potential improvements in inner retinal function and visual field parameters, including visual field mean deviation (MD), although findings remain limited and heterogeneous [5,6].

Given the global burden of glaucoma and limitations of current therapies, interest in nicotinamide is increasing. This systematic review aims to evaluate the efficacy of nicotinamide in maintaining MD in adults with glaucoma, alongside its safety profile and broader ophthalmological outcomes, to clarify its potential as a disease-modifying intervention.

## Review

Materials and methods

This systematic review was undertaken in accordance with the PRISMA 2020 framework to ensure transparency and comparability [7]. Eligibility criteria were defined using the patient, intervention, comparator, outcome, study design, timeframe (PICOST) structure. Studies were included if they recruited adults (18 years old or older) diagnosed with any form of glaucoma, evaluated an intervention including nicotinamide therapy against any relevant comparator, reported MD as either a primary or secondary outcome, and were designed as randomized controlled trials (RCTs). Pediatric populations were excluded to minimize inclusion of rare glaucoma subtypes that could reduce generalizability. Studies involving ocular hypertension were excluded due to the absence of established glaucomatous optic neuropathy, which may confound interpretation of visual field outcomes. Given the predominance of preclinical and observational data identified during scoping, only RCTs were included to maximize clinical applicability. The inclusion and exclusion criteria are detailed in Table 1.

**Table 1:** PICOST criteria for inclusion and exclusion IOP: intraocular pressure; MD: mean deviation; PICOST: patient, intervention, comparator, outcome, study design, timeframe; RCT: randomized controlled trial

Criteria	Included	Excluded
Population	Participants have a diagnosis of glaucoma; participants are adults (18 years old or older)	Children under the age of 18
Intervention	Any dose or regimen involving nicotinamide with or without standard IOP-lowering therapy or other interventions	Other therapy regimens not involving nicotinamide
Comparator	Any relevant comparator (placebo, standard IOP-lowering therapy) in participants with glaucoma	Comparison only with healthy participants
Outcome	MD as a reported primary or secondary outcome	MD not reported
Study design	Any RCT including at least one intervention arm and at least one control arm	Preclinical/basic science studies, letters, editorials, abstracts, animal studies, cohort studies, systematic reviews
Timeframe	Any publication date	No restrictions
Publication type	English language	Foreign language without translation, inaccessible via databases, and no response from authors upon request

MD was chosen as the primary outcome given its well-established use as a global measure of visual field loss in standard automated perimetry (SAP). It is related to RGC function and patient-reported quality of life, and it is regarded as a meaningful endpoint in trials assessing neuroprotective strategies, correlating with Food and Drug Administration-approved endpoints [8,9]. Secondary outcomes included electroretinogram (ERG) measures and additional perimetric indices such as pattern standard deviation (PSD) and visual field index (VFI), alongside IOP and optical coherence tomography (OCT) parameters. Data on adverse events and treatment tolerability were also collected.

A systematic search of MEDLINE, Embase, ClinicalTrials.gov, and the Cochrane Library was conducted in March 2026. No date restrictions were applied; however, non-English publications were excluded. Full search strategies are detailed in Appendix A.

Study selection was conducted independently by two reviewers using a three-stage process comprising title, abstract, and full-text screening. Disagreements were resolved through discussion with a third author to reach a consensus. Studies were excluded if they were non-randomized, conference abstracts, case reports, reviews, or basic science investigations.

Data extraction was performed independently by two reviewers using Rayyan software (Rayyan Systems, Inc., Cambridge, MA, USA) [10]. Extracted data included study design, population characteristics, intervention details, and outcomes relevant to efficacy and risk of bias. A standardized, pilot-tested data collection framework was implemented in Microsoft Excel (Microsoft Corporation, Redmond, WA, USA), with discrepancies resolved by consensus with a third author. A change of 1 dB in MD over one year was defined as clinically significant [11].

Risk of bias was assessed using the Risk of Bias 2 (RoB 2) tool, evaluating multiple domains of potential bias [12]. Studies were classified as low risk, some concerns, or high risk of bias.

Meta-analysis was deemed infeasible due to substantial heterogeneity in populations, interventions, outcome reporting, and follow-up identified during the scoping review. Inconsistent visual outcome reporting and analytical variability limited comparability. Findings were therefore synthesized narratively with descriptive statistics, including means and 95% CIs, in accordance with PRISMA 2020 recommendations [7]. Results are presented as differences in MD between intervention and comparator groups in a summary of findings table, following Cochrane Collaboration guidance [13]. Certainty of evidence was appraised using the Grading of Recommendations Assessment, Development and Evaluation (GRADE) approach by two independent reviewers, with consensus reached via a third author [14].

Results

**Figure 1:**
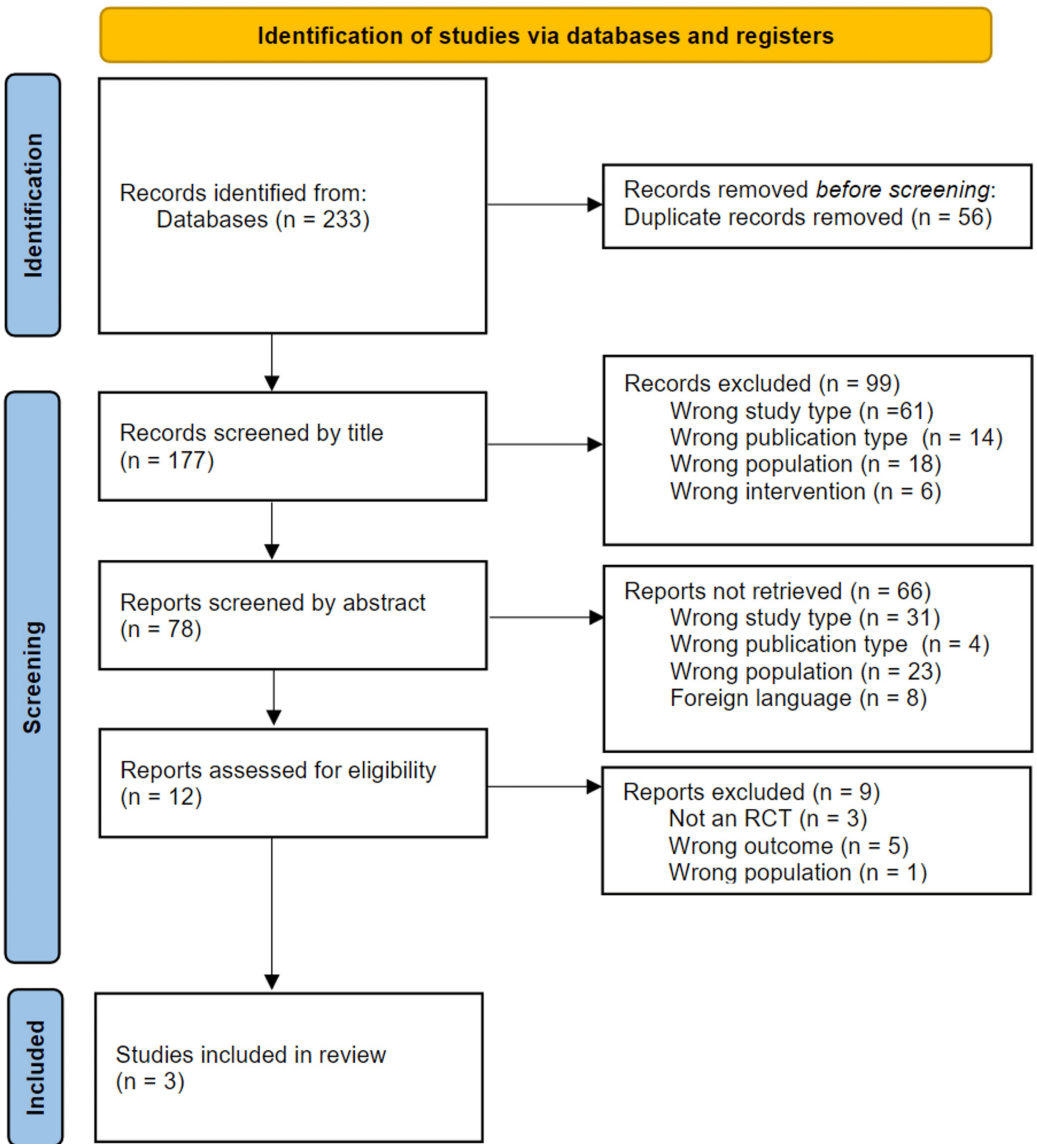
PRISMA 2020 flow diagram of study selection

Figure 1 shows the PRISMA flow diagram of the study selection process. The systematic search identified 233 records. After removal of 56 duplicates, 99 articles were excluded during title screening, and a further 66 were excluded after abstract screening. Twelve studies proceeded to full-text review, of which three met the inclusion criteria and were included in the final analysis.

Study Characteristics

In total, data from 152 participants were included across the three eligible studies. The first trial’s results were reported in 2020 by Hui et al., a phase II RCT utilizing a crossover design without washout in Australia [15]. In 2022, De Moraes et al. published their findings from a phase II RCT parallel-arm study conducted in the USA, utilizing an intervention regimen involving both nicotinamide and pyruvate [16]. Finally, Ha et al. published another phase II RCT crossover study in 2026, investigating nicotinamide in normal-tension glaucoma in South Korea [17]. Table 2 shows the characteristics of the included studies. All trials allowed for concurrent IOP-lowering therapy in both arms, and in all trials, the comparator was placebo.

**Table 2:** Characteristics of included studies ERG: electroretinography; IOP: intraocular pressure; MD: mean deviation; PSD: pattern standard deviation; RCT: randomized controlled trial; RNFL: retinal nerve fiber layer; SITA: Swedish Interactive Thresholding Algorithm; VFI: visual field index

Reference	Design	Country	Population	Intervention (with standard IOP-lowering therapy)	Primary outcome, relevant ophthalmological secondary outcomes	Number of participants	Length of follow-up
Hui et al. (2020) [15]	Phase II RCT crossover without washout	Australia	Glaucoma	Nicotinamide (1500 mg/day for six weeks, then 3000 mg for six weeks)	Full-field ERG, MD, PSD, VFI, RNFL	57	24 weeks (12 weeks on each arm)
De Moraes et al. (2022) [16]	Phase II RCT parallel arm	USA	Open-angle glaucoma	Nicotinamide (ascending doses 1000 mg/day to 3000 mg over three weeks) and pyruvate (ascending dose from 1500 mg/day to 3000 mg) over two months	Number of improved visual field test locations (24-2 SITA program), MD, PSD, VFI, RNFL	42	Median 2.2 months (2.0-2.4 months)
Ha et al. (2026) [17]	Phase II RCT crossover without washout	South Korea	Normal-tension glaucoma	Nicotinamide (1000 mg/day for six weeks, then 2000 mg for six weeks)	Full-field ERG, MD, PSD, VFI	53	24 weeks (12 weeks on the arm)

MD

Nicotinamide was not found to significantly change rates of MD decline in any of the three trials. In Hui et al.’s RCT, mean MD did improve by +0.10 dB compared to placebo across the 24-week trial but did not reach statistical significance (95% CI -0.33 to +0.53 dB, p = 0.63) [15]. De Moraes et al. reported no significant effect on MD upon univariable logistic regression modeling with nicotinamide and pyruvate as the predictor variable, with a reduction in mean MD of -0.08 dB/week in the intervention group compared to placebo (95% CI -0.27 to +0.09, p = 0.36) [16]. Finally, Ha et al. demonstrated small median MD improvements in both placebo and intervention arms, which were reported graphically, and no significant difference was noted (p value not reported) [17]. These findings are summarized in Table 3.

**Table 3:** Summary of findings GRADE: Grading of Recommendations Assessment, Development and Evaluation; MD: mean deviation

Intervention (reference)	Control	Outcome (MD)	Comparison between intervention and control	Certainty of evidence (GRADE)
Nicotinamide (1500 mg for six weeks, 3000 mg for six weeks) [15]	Placebo	Duration: 24 weeks (12-week crossover). Control arm: mean MD deteriorated in the placebo. Intervention arm: mean MD improved by +0.10 dB (95% CI -0.33 to 0.53) compared to placebo.	No significant difference vs control, p = 0.63	⊕⊕ΟΟ Low
Nicotinamide and pyruvate (ascending doses) [16]	Placebo	Duration: two months. Control arm: mean MD not given; used a mixed-effects model and reported results with treatment as a univariable predictor of MD change. Intervention arm: no significant effects found upon modeling; difference of -0.08 dB/week compared to placebo (95% CI -0.27 to 0.09).	No significant difference vs control, p = 0.36	⊕⊕ΟΟ Low
Nicotinamide (1000 mg for six weeks, 2000 mg for six weeks) [17]	Placebo	Duration: 24 weeks (12-week crossover). Control arm: median MD showed a small improvement in the figure. Intervention arm: median MD showed a small improvement in the figure.	No significant difference vs control, p-value not given	⊕ΟΟΟ Very low

Secondary Outcomes

The primary outcome for the two crossover studies by Ha et al. and Hui et al. was full-field ERG. Both studies reported significant improvements in ERG metrics. Ha et al. reported significant improvements in both b-wave amplitude (representing bipolar cell function) and photopic negative response (PhNR) amplitude (representing RGC function) following nicotinamide treatment. The mean b-wave amplitude was 2.214 μV higher in the nicotinamide group than in the placebo group (p = 0.045), and the mean PhNR amplitude was 1.807 μV higher (p = 0.032) [17]. Hui et al. reported significant improvement in mean Vmax (maximum predicted response amplitude) and PhNR Vmax following nicotinamide. Mean Vmax improved by 12.6% in the treatment group vs 3.6% in the placebo group after 12 weeks (p = 0.03), and mean PhNR Vmax improved by 14.8% following nicotinamide vs 5.2% in the placebo group (p = 0.04) [15].

The primary outcome for the parallel-arm trial investigating nicotinamide and pyruvate was the number of visual field test locations improving beyond normal variability on 24-2 SAP. Nicotinamide and pyruvate were reported to significantly increase the number of such locations compared to placebo (median 15 sites with nicotinamide, seven without, p = 0.005) [16].

PSD was reported in all three studies as another parameter assessed on SAP. Significant improvement was reported by De Moraes et al. on mixed-effects modeling following nicotinamide/pyruvate treatment, with an improvement of -0.06 dB/week with treatment compared to +0.02 dB/week deterioration on placebo (p = 0.02) [16]. However, the two crossover studies found no significant differences in PSD after nicotinamide for 12 weeks [15,17].

Two of the studies reported VFI; neither De Moraes et al.’s parallel-arm RCT nor Ha et al.’s crossover study reported significant changes following treatment. De Moraes et al. and Hui et al. reported retinal nerve fiber layer thickness findings following OCT, and no significant differences were found following treatment for this outcome. IOP was reported by Hui et al., with no significant differences between the nicotinamide and placebo arms. Further information on secondary outcomes is presented in Table 4 [15-17].

**Table 4:** Secondary outcomes COR: coefficient of repeatability; ERG: electroretinography; IOP: intraocular pressure; PhNR: photopic negative response; PSD: pattern standard deviation; RNFL: retinal nerve fiber layer; SITA: Swedish Interactive Thresholding Algorithm; VFI: visual field index

Reference	PSD	Secondary outcome 2	Secondary outcome 3	Secondary outcome 4
Hui et al. (2020) [15]	No significant changes in PSD, difference of -0.25 dB between treatment and placebo groups (95% CI -0.63-0.14), p = 0.20.	IOP: No significant differences between placebo and treatment arms. Mean IOP was 13.8, SD 4.1 mmHg following treatment and 13.4, SD 2.4 mmHg after placebo, p = 0.59.	ERG: Significant improvement in mean Vmax after treatment, with a mean 12.6% improvement in the treatment group (95% CI 5.0-20.2) vs 3.6% (95% CI -3.4-10.5) in the placebo, p = 0.03. Significant improvement in mean PhNR Vmax after treatment, with 14.8% improvement in Vmax in the treatment group (95% CI 2.8-26.9) compared to 5.2% in the placebo (95% CI -4.2-14.6), p = 0.04. Primary outcome of this study.	RNFL: No significant differences between treatment and placebo groups. Treatment group mean change -0.3, SD 2.9 μm vs placebo 0.4, SD 2.4 μm, p = 0.11.
De Moraes et al. (2022) [16]	Mixed-effects model showed significant PSD improvement in treatment group -0.06 dB/week (95% CI -0.30 to 0.06) vs placebo 0.02 dB/week (95% CI -0.07 to 0.07), p = 0.02.	VFI: No differences in VFI rate of change. Treatment group median change 0.09% per week (-0.4-0.6) vs placebo -0.02% (-0.2-0.1), p = 0.71.	Number of SITA 24-2 sites improved: significant improvement in treatment group (median 15, IQR 6-25) vs placebo (median 7, IQR 6-11), p = 0.005. Primary outcome of this study.	RNFL: No differences in rate of RNFL thickness change (difference between treatment and placebo 0.18, 95% CI -0.06 to 0.43, p = 0.13).
Ha et al. (2026) [17]	No significant difference between arms, p-value not provided. Mild improvements in median PSD noted in both arms (less than 1 dB after 12 weeks).	VFI: No significant differences between arms, p-value not provided. Median was 0% change in the treatment arm and between 0 and -1% in the placebo arm after 12 weeks.	ERG: Significant improvement in mean PhNR amplitude in treatment group (3.121 μV, 95% COR ± 3.968) vs placebo (0.996 μV, 95% COR ± 4.190), p = 0.045. Significant improvement in mean b-wave amplitude in treatment group (2.112 μV, 95% COR ± 3.220) vs placebo (0.305 μV, 95% COR ± 3.279), p = 0.032. Primary outcome of this study.	None reported

Safety and Adverse Events

No serious adverse events were recorded in any of the three trials, including acute liver injury. Some mild adverse events, most commonly gastrointestinal discomfort, were reported across the trials with nicotinamide [15-17].

Risk of Bias Appraisal

Table 5 presents the risk of bias assessment using the RoB 2 tool. All studies had some concerns for the overall risk of bias due to loss to follow-up. The crossover trials did not utilize a washout period, introducing potential confounding. In comparison, De Moraes et al. reported MD using a model without reporting descriptive statistics, preventing conclusions on this outcome from being drawn from the original dataset and instead relying on the authors’ model [15-17].

**Table 5:** RoB 2 scores of included studies RoB 2: Risk of Bias 2

Reference	RoB from random sequence generation	RoB from deviations from the intended procedure	RoB from missing outcome data	RoB from measuring the outcome	RoB from the selection of reported results	Overall RoB
Hui et al. (2020) [15]	Low	Some concerns (crossover design without washout)	Some concerns (>5% loss to follow-up)	Low	Low	Some concerns
De Moraes et al. (2022) [16]	Low	Low	Some concerns (>5% loss to follow-up)	Some concerns (univariable modeling not reporting descriptive statistics)	Low	Some concerns
Ha et al. (2026) [17]	Low	Some concerns (crossover design without washout)	Some concerns (>5% loss to follow-up)	Low	Low	Some concerns

Certainty of Evidence

Table 6 presents the certainty of evidence behind each intervention according to the GRADE tool. All studies framed a clear research question and provided a direct answer to the question of this review. All trials scored low due to being downgraded for some concerns in the RoB assessment and small sample size. Ha et al.’s intervention regimen was further downgraded by one point due to only reporting MD graphically without descriptive statistics [15-17].

**Table 6:** GRADE scores of certainty of evidence for included interventions GRADE: Grading of Recommendations Assessment, Development and Evaluation; RCT: randomized controlled trial

Intervention	Risk of bias	Inconsistency	Indirectness	Imprecision	Publication bias	Other factors	Overall quality of evidence
Nicotinamide (1500 mg for six weeks, then 3000 mg for six weeks) [15]	Some concerns (-1)	None - 1 RCT (0)	None - answers review question directly (0)	Small sample size (-1)	None detected (0)	None (0)	⊕⊕ΟΟ Low
Nicotinamide and pyruvate (ascending doses) [16]	Some concerns (-1)	None - 1 RCT (0)	None - answers review question directly (0)	Small sample size (-1)	None detected (0)	None (0)	⊕⊕ΟΟ Low
Nicotinamide (1000 mg for six weeks, then 2000 mg for six weeks) [17]	Some concerns (-1)	None - 1 RCT (0)	None - answers review question directly (0)	95% CI does encompass clinically significant results but are graphically reported. P values not reported. Small sample size (-2)	None detected (0)	None (0)	⊕ΟΟΟ Very low

Discussion

Current evidence does not support nicotinamide in improving MD in adults with glaucoma (Table 3). All included trials evaluated high-dose nicotinamide using escalating dose regimens of 2000-3000 mg daily, substantially higher than typical supplement doses of 500-1000 mg [18]. As adults with glaucoma are known to have reduced serum nicotinamide levels, these regimens were intended to restore rather than maintain physiological levels [4]. However, all studies were limited by short follow-up durations (maximum 24 weeks) and small sample sizes. De Moraes et al.’s trial’s primary endpoint involved data derived from SAP; hence, to increase statistical power, they utilized the “wait-and-see” approach, which clusters visual field tests at the beginning and end of trials [16]. This strategy increases power by reducing the effect of intra-trial variability on MD slope estimates and was verified on a large dataset from the UK Glaucoma Treatment Study [19]. However, this trial only recruited 42 people and analyzed 32. Furthermore, this strategy is better for detecting rapid visual field loss and will not address more gradual changes [20].

All three trials met their primary outcomes, demonstrating improvements in ERG parameters in the crossover studies and increased numbers of improving visual field locations in the nicotinamide/pyruvate trial. The ERG parameters assessed, particularly b-wave and PhNR amplitudes, are considered early markers of inner retinal and RGC function. Improvements in these electrophysiological measures are therefore consistent with the proposed mechanism of neuroprotection. Additionally, the greater number of improving visual field locations observed following nicotinamide and pyruvate treatment raises the possibility that these neuronal improvements may translate into functional visual field benefits.

However, these findings should be interpreted with caution due to significant limitations, including loss to follow-up, small sample sizes, short follow-up durations, and potential risk of bias. Furthermore, two studies used crossover designs without washout periods. The number of improving visual field locations is also an unconventional outcome measure in studies assessing SAP, where MD and PSD remain the established gold-standard metrics. Given these limitations and the resulting low GRADE certainty ratings, the findings should be regarded as promising but primarily hypothesis-generating. These phase II trials provide hypothesis-generating data meriting further investigation of nicotinamide as a potential neuroprotective therapy but not conclusive evidence that can impact practice.

The current body of literature comprises a limited number of contemporary early-phase RCTs, reflecting growing interest in nicotinamide as a neuroprotective strategy. Definitive conclusions regarding its impact on MD and other clinically meaningful ophthalmic outcomes will require larger, adequately powered phase III studies. In a 2025 position statement, the American Glaucoma Society and American Academy of Ophthalmology advised that nicotinamide remains unapproved, with insufficient evidence regarding both efficacy and safety for routine use. This is partly due to the potential severe complication of drug-induced liver injury in people taking high-dose nicotinamide [18]. Their guidance recommends monitoring liver function for doses below three grams/day, while doses exceeding three grams/day should be restricted to clinical trial settings [21]. These recommendations align with the conclusions of this review, which finds the current evidence base too limited to inform clinical practice.

Ongoing and Future Research

Interest in nicotinamide continues to expand, with several ongoing and recent studies contributing additional insights. Two relevant trials that did not assess MD still provided data supporting further investigation. A 2025 RCT involving 58 participants evaluated a 500 mg supplementation regimen and demonstrated improvements in quality of life, with statistically significant gains in Glaucoma Quality of Life-15 scores. Benefits were observed across multiple domains, including central and near vision, peripheral vision, and dark adaptation [22]. Additionally, a 2023 prospective study involving 120 participants (30 healthy controls and 90 with glaucoma) assessed the effects of 500 mg nicotinamide over two weeks, reporting increased perfusion density at the optic nerve head and in the temporal macula [23]. Although these studies remain limited in scale and duration, they provide early indications of potential benefits in patient-reported outcomes and ocular perfusion, warranting further exploration in larger trials.

Several ongoing trials are investigating the neuroprotective efficacy of nicotinamide globally (NCT05695027, NCT06991712, NCT05275738, NCT05405868, and NCT07006194). Most of these trials are investigating high-dose nicotinamide in isolation; one is investigating four NAD+ precursors, including nicotinamide (NCT06991712), and one is investigating nicotinamide with pyruvate (NCT05695027). With a target cumulative recruitment of over 1000 participants across these trials, the results will contribute significantly to evidence-based practice and justification of a large phase III trial [24].

This review highlights the need for further trials. Ideally, future trials would occur over an extended timeframe with larger populations to provide greater statistical power than the current proof-of-concept phase II trials. There was significant heterogeneity in the way MD was reported, making interpretation difficult when synthesizing results across multiple trials. This could be remedied in future and ongoing trials by providing descriptive statistics of MD change per unit time, ideally per year, for long-term conditions like glaucoma [11]. Trials should also follow standardized reporting guidelines such as the CONSORT protocol to allow for appraisal of results [25]. Utilizing these recommendations, future meta-analyses could be conducted to quantitatively compare intervention efficacy.

Limitations

Given the early stage of the evidence base examining nicotinamide’s impact on MD, this review cannot draw firm conclusions regarding the safety or efficacy of its neuroprotective effects. Further research is needed. Nonetheless, it provides a clear summary of the current preliminary literature, highlights emerging trends, and outlines key considerations for the design of future phase III studies. A limitation of this review is the absence of meta-analysis, driven by substantial heterogeneity in MD change reporting, secondary endpoints, study populations, and interventions. Although a narrative approach enables more in-depth qualitative comparison, it does not offer the quantitative estimates that would better inform clinicians and researchers.

## Conclusions

Nicotinamide has emerged as a potential neuroprotective therapy in glaucoma and is the subject of ongoing clinical investigation. However, current evidence does not demonstrate improvement in MD, despite reported benefits in ERG parameters and the number of improved visual field locations. These findings remain preliminary, limiting the reliability of conclusions drawn. Accordingly, this review aligns with current guidance that does not support the use of nicotinamide in clinical practice. Overall, there is insufficient evidence to support its role in preserving MD in glaucoma, underscoring the need for larger, more robust phase III studies.

## Appendices

Appendix A 

**Table 7:** Search strategy

Database	Search strategy	Number of hits
MEDLINE	#1 exp glaucoma/ [MeSH Terms] #2 glaucoma/ [Title/Abstract/Keyword] #3 nicotinamide/ [MeSH Terms] #4 nicotinamide [Title/Abstract] #5 #1 OR #2 #6 #3 OR #4 #7 #5 AND #6	43
Embase	#1 exp nicotinamide/ #2 nicotinamide*.ti. or nicotinamide*.ab. #3 1 or 2 #4 exp glaucoma/ #5 glaucoma.ti. or glaucoma.ab. #6 4 or 5 #7 3 and 6	166
Cochrane Library	#1 nicotinamide: ti,ab,kw #2 MeSH descriptor: [ glaucoma] explode all trees #3 (glaucoma):ti,ab,kw #4 (#1) and (#2 or #3)	20
ClinicalTrials.gov	‘glaucoma/’ condition AND ‘nicotinamide/’ treatment Filter trial status: completed	4
